# Development of the CO_2_-Resistant Gel by Designing a Novel CO_2_-Responsive Polymer for Channel Control in Low-Permeability Reservoirs

**DOI:** 10.3390/gels12010057

**Published:** 2026-01-07

**Authors:** Xiangjuan Meng, Xinjie Xu, Yining Wu, Zhenfeng Ma, Herui Fan, Ziyi Wang, Wenhao Ren, Zhongzheng Xu, Mingwei Zhao

**Affiliations:** 1 State Key Laboratory of Deep Oil and Gas, China University of Petroleum (East China), Qingdao 266580, Chinaxuxinjie1028@163.com (X.X.);; 2 Shandong Key Laboratory of Oil and Gas Field Chemistry, China University of Petroleum (East China), Qingdao 266580, China

**Keywords:** CO_2_-responsive polymer, CO_2_-resistant gel, gas channeling control

## Abstract

To address the problem of serious gas channeling during CO_2_ flooding in low-permeability reservoirs, which leads to poor oil recovery, this study developed a CO_2_-resistant gel using a novel CO_2_-responsive polymer (ADA) for gas channel control. The ADA polymer was synthesized via free-radical copolymerization of acrylamide (AM), dimethylaminopropyl methacrylamide (DMAPMA), and 2-acrylamido-2-methylpropanesulfonic acid (AMPS), which introduced protonatable tertiary-amine groups and sulfonate moieties into the polymer backbone. Comprehensive characterizations confirmed the designed structure and adequate thermal stability of the ADA polymer. Rheological tests demonstrated that the ADA polymer solution exhibits significant CO_2_-triggered viscosity enhancement and excellent shear resistance. When crosslinked with phenolic resin, the resulting ADA gel showed outstanding CO_2_ tolerance under simulated reservoir conditions (110 °C, 10 MPa). After 600 s of CO_2_ exposure, the ADA gel retained over 99% of its initial viscosity, whereas a conventional HPAM-based industrial gel degraded to 61% of its original viscosity. The CO_2_-resistance mechanism involves protonation of tertiary amines to form quaternary ammonium salts, which electrostatically interact with sulfonate groups, creating a reinforced dual-crosslinked network that effectively protects the gel from H^+^ ion attack. Core flooding experiments confirmed its ability to enhance oil recovery by plugging high-permeability channels and diverting flow, achieving a final recovery of up to 48.5% in heterogeneous cores. This work provides a novel gel system for improving sweep efficiency and storage security during CO_2_ flooding in low-permeability reservoirs.

## 1. Introduction

With the ongoing advancement of oil and gas exploration and development, unconventional resources such as low-permeability, ultra-low-permeability, and shale reservoirs are accounting for an increasingly significant proportion of production [1,2]. The proportion of newly discovered reserves in such reservoirs continues to rise annually. However, in the case of relying solely on natural energy for development, oil recovery in low-permeability reservoirs is extremely low (typically below 10%) [3,4]. Therefore, it is necessary to inject water or gas into the reservoir to supplement energy and enhance oil recovery. Due to the small pore throats characteristic of low-permeability reservoirs, water injection often leads to rapidly increasing pressure of the reservoir, significantly reduced injectivity, or even complete injection failure, thereby limiting the effectiveness of water flooding [5,6]. In contrast, CO_2_ can more readily penetrate micro-scale and nano-scale pores. In addition to replenishing reservoir energy, CO_2_ can effectively displace light- to medium-crude oil from the reservoir, significantly improving recovery. The advantages of CO_2_, including viscosity reduction, volume expansion, interfacial tension reduction, and light hydrocarbon extraction, make it an attractive enhanced oil recovery (EOR) agent for low-permeability reservoirs [7]. Moreover, the use of CO_2_ in EOR aligns with carbon capture, utilization, and storage (CCUS) objectives, highlighting its environmental significance [8].

Hydraulic fracturing is essential for the efficient development of low-permeability reservoirs due to their poor permeability. However, the resulting fracture-matrix dual-medium system exhibits strong heterogeneity [9]. Under reservoir conditions, the viscosity of supercritical CO_2_ is much lower than that of crude oil and formation water, which can lead to severe channeling through fractures. This results in ineffective cycling of CO_2_, reduced sweep efficiency, and ultimately diminished oil recovery and CO_2_ storage performance. Therefore, controlling gas channeling represents a major technical challenge in CO_2_ flooding for low-permeability reservoirs [10,11,12].

To address CO_2_ breakthrough in low-permeability reservoirs, two main categories of technologies have been developed: water-alternating-gas (WAG) injection and chemical-based conformance control [13,14,15,16]. WAG involves alternating injections of water and CO_2_, which increases the effective viscosity of the CO_2_ phase, reduces relative permeability and stabilizes the displacement front, thereby improving sweep efficiency. WAG techniques include miscible, immiscible, and hybrid modes [17,18,19]. Since residual oil saturation after gas flooding is generally lower than that after water flooding, WAG may further reduce remaining oil saturation in three-phase regions, enhancing microscopic displacement efficiency. First applied in sandstone reservoirs in Alberta, Canada in the 1960s, WAG has since been widely adopted in the United States and Canada, with about 80% of CO_2_-WAG projects reported as successful [20]. Recent variants include nanofluid-assisted WAG, low-salinity WAG, polymer/surfactant-enhanced WAG, and foam-assisted WAG [21]. While WAG typically improves oil recovery by 5–10%, its application in low-permeability reservoirs is often limited by poor injectivity and water blockage due to fine pore throats and low connectivity. Moreover, WAG shows limited effectiveness in highly heterogeneous or fractured formations [22].

Chemical-based conformance control agents include thickening polymers, foams, alkaline solutions (which generate precipitates), particles, and gels. Thickening polymers dissolved in supercritical CO_2_ can increase its viscosity. However, effective polymers, typically silicon-based or fluorine-based, face challenges such as poor solubility, high cost, and environmental concerns, hindering field-scale application [23,24]. CO_2_ foam can improve sweep efficiency by increasing flow resistance in high-permeability channels of the heterogeneous reservoir via the Jamin effect, but its use in tight formations often leads to excessively high injection pressures and difficulties in delivering foaming agents deep into the reservoir [25,26]. Alkaline solutions react with CO_2_ to form precipitates that block high-permeability layers, but controlling pH under complex reservoir conditions remains difficult [27]. Particulate agents such as pre-crosslinked gel particles and polymer microspheres plug channels through swelling or bridging, but they often exhibit rapid expansion and poor deep migration [28]. Gels are widely used for CO_2_ channeling control, which can form plugs in the breakthrough flow channel, forcing CO_2_ to turn to the low-permeability areas. Syed et al. [29] demonstrated that gels formed by cross-linking polyacrylamide with chromium (III) could effectively block CO_2_ in high-permeability sandstone and fractured carbonate models at 40 °C, achieving a reduction in CO_2_ permeability exceeding 90%. Similarly, Durucan et al. [30] reported that a gel system based on polyacrylamide cross-linked with zirconium reduced CO_2_ permeability by 99.87% in core experiments at 40 °C. Crosslinked polymer gels, in particular, allow adjustable crosslinking time for deep placement [31,32]. However, under reservoir temperature and pressure, supercritical CO_2_ acidifies the environment to a pH of 2–4 and exerts physical degradation, demanding exceptional CO_2_ resistance from gel systems [33,34,35].

In this study, to meet the requirements for in-depth conformance control in low-permeability reservoirs undergoing CO_2_ flooding, a CO_2_-responsive polymer was synthesized by introducing CO_2_-responsive monomers, and a CO_2_-resistant gel was formulated using a phenolic prepolymer crosslinker. The rheological behavior and microstructure of the gel under CO_2_ conditions were investigated to elucidate its stability mechanism in low-permeability reservoirs. This work contributes to improving CO_2_ flooding efficiency and enhancing CO_2_ utilization and storage, demonstrating significant potential for field application.

## 2. Results and Discussion

**Figure 1:**
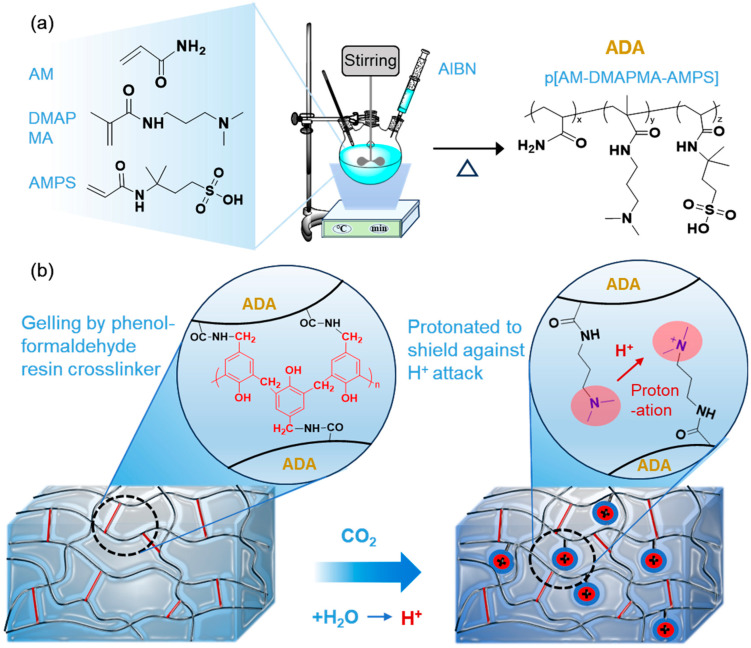
(**a**) Schematic diagram of the synthetic route and molecular structure of the CO_2_-responsive polymer ADA; (**b**) Schematic diagram of the crosslinked structure and the CO_2_ resistance mechanism of the CO_2_-resistant gel.

As shown in Figure 1a, the design of the CO_2_-resistant gel is based on the preparation of a CO_2_-responsive polymer to ensure its stability in a CO_2_ environment. To achieve the desired performance, a CO_2_-responsive polymer (ADA) containing protonatable groups (-(R_2_)N), crosslinking groups (-NH_2_), temperature-tolerant and salt-tolerant groups (-SO_3_^−^) was synthesized via free radical copolymerization in an aqueous solution [36]. Three monomers were selected: AM, DMAPMA, and AMPS. This novel polymer was subsequently characterized, and its rheological properties were investigated, laying the foundation for the subsequent preparation of the CO_2_-resistant gel. The primary damaging mechanism of CO_2_ to polyacrylamide gels is the formation of a strongly acidic environment when CO_2_ reacts with water, where a high concentration of H^+^ ions can accelerate the hydrolysis of crosslinking sites (amide groups), thereby weakening the gel network strength. The tertiary amine groups in the CO_2_-responsive polymer become protonated in this acidic environment, converting into quaternary ammonium salts and imparting a strong positive charge to the side chains [37]. As illustrated in Figure 1b, these positively charged side chains can shield the crosslinking sites from attack by H^+^ ions, thereby protecting the gel structure and enabling CO_2_ resistance [38,39].

### 2.1. Chemical Structure Characterization of the ADA Polymer

**Figure 2:**
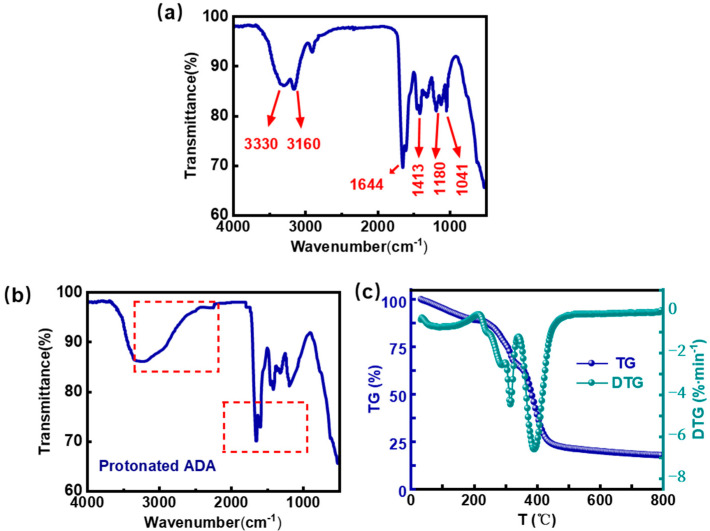
The FTIR spectrums of CO_2_-responsive polymer ADA before (**a**) and after (**b**) protonation by CO_2_, (**c**) TGA spectrum of the CO_2_-responsive polymer ADA.

Figure 2a presents the FTIR spectrum of the synthesized CO_2_-responsive polymer ADA. The absorption peaks observed at 3330 cm^−1^, 1644 cm^−1^, and 1413 cm^−1^ were assigned to the stretching vibration of -NH_2_, the stretching vibration of -C=O, and the bending vibration of C-N in the amide group, respectively. The results confirmed the successful introduction of the key crosslinking group (–CONH_2_). The peak at 3160 cm^−1^ corresponded to the stretching vibration of -NH, while the peak at 1180 cm^−1^ was attributed to the asymmetric stretching vibration of S=O, evidencing the successful incorporation of the sulfonic acid group (-SO_3_^−^), which enhances the polymer’s hydrophilicity and stability. Additionally, the absorption peak at 1041 cm^−1^ was ascribed to the C-N stretching vibration in the tertiary amine group, verifying the successful introduction of the CO_2_-responsive monomer (DMAPMA). Analysis of the characteristic absorption peaks in the FTIR spectrum indicated that the functional group composition of the synthesized polymer is consistent with the designed structure. Additionally, as shown in Figure 2b, the FTIR spectrum of the ADA polymer protonated by CO_2_ is analyzed. The most significant evidence is the characteristic broad-strong absorption uplift caused by the N^+^-H stretching vibration of the quaternary ammonium cation in the region of ~2400–2800 cm^−1^. At the same time, the shoulder peak in the region of ~1550–1680 cm^−1^ is due to the superposition of the carbonyl peak between the carbamate group generated after protonation and the amide group of the ADA polymer.

There is usually a high reservoir temperature in oilfield development, which also puts forward the requirement of temperature resistance for the gel used for gas channel control. A polymer with good thermal stability is fundamental to ensuring the temperature resistance of the gel. In this study, the thermal degradation characteristics of the synthesized CO_2_-responsive polymer (ADA) were investigated by thermogravimetric analysis (TGA). The experimental results are shown in Figure 2c. According to the TGA curve, the polymer exhibited distinct mass loss characteristics in three stages during heating, corresponding to the following temperature ranges: Stage I: 35–214 °C, Stage II: 214–366 °C, and Stage III: above 366 °C. In Stage I, the mass loss was only 12.1%, primarily due to the evaporation and decomposition of adsorbed water. The maximum mass loss rate was merely 0.92%·min^−1^, indicating that the molecular structure of the synthesized ADA polymer remained stable without decomposition within this temperature range. Above 215 °C, ester, amide, and sulfonic acid groups in the side chains of the ADA polymer began to decompose significantly. In the temperature range of 215–366 °C, the polymer lost 39.71% of its mass, with a maximum mass loss rate of 4.47%·min^−1^ at 316 °C. The mass loss in the final heating stage (Stage III) was mainly attributed to the cleavage of the C–C backbone. At the end of the test, the residual mass of the synthesized polymer was 17.59% of its initial mass. Since reservoir temperatures in most CO_2_ flooding projects generally do not exceed 200 °C, the TGA results confirm that temperatures below 214 °C do not affect the molecular structure of the synthesized ADA polymer. It is concluded that the ADA polymer fully meets the thermal stability requirements for in-depth conformance control in CO_2_ flooding operations.

### 2.2. CO_2_ Responsiveness of the ADA Polymer

**Figure 3:**
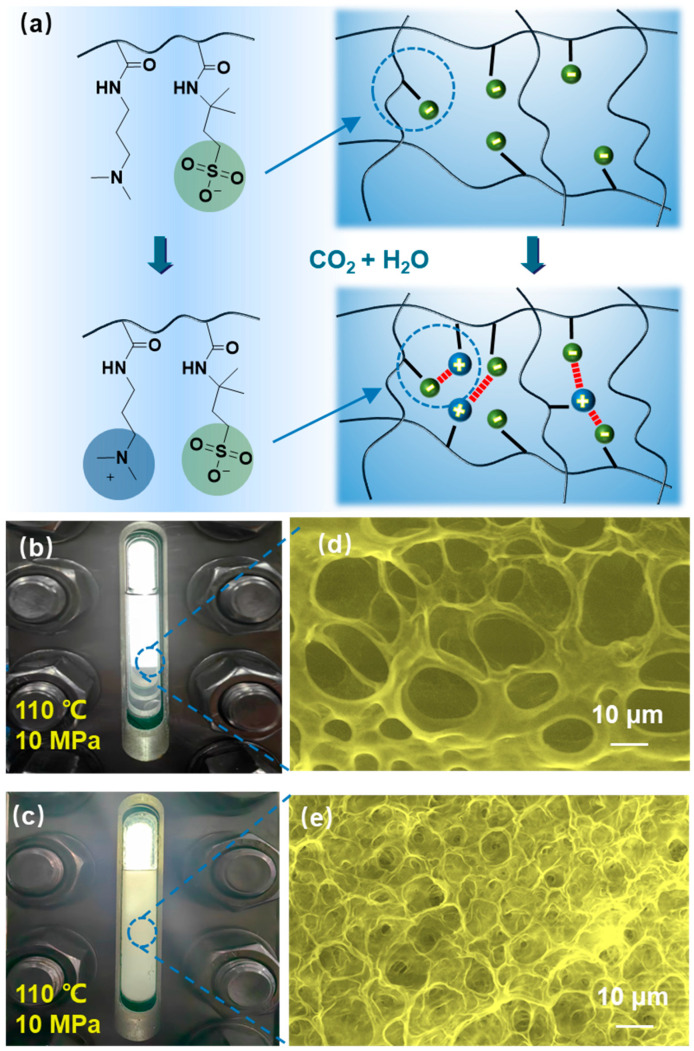
(**a**) The protonation schematic of the CO_2_-responsive polymer ADA; Physical images (**b**,**c**) and ESEM images (**d**,**e**) of the ADA polymer before (**b**,**d**) and after (**c**,**e**) response to supercritical CO_2_.

As illustrated in Figure 3a, the AMPS groups in the synthesized ADA polymer readily hydrolyze in aqueous solution to form sulfonate moieties. This not only enhances the water solubility of ADA polymers, but also introduces electrostatic repulsion between polymer chains, promoting a more extended conformation in solution. Upon CO_2_ introduction, the solution becomes acidic, protonating the tertiary amine groups in ADA to form positively charged quaternary ammonium salts. Electrostatic interactions between these quaternary ammonium groups and the sulfonate groups then establish a physically crosslinked network. The CO_2_-response mechanism of the ADA polymer was verified through macroscopic simulation experiments and environmental scanning electron microscopy (ESEM) imaging. Macroscopic simulation results under reservoir-relevant conditions (110 °C, 10 MPa, where CO_2_ is in a supercritical state) are presented in Figure 3b,c. Before the CO_2_ injection, the ADA polymer solution remained clear and transparent (Figure 3b). After CO_2_ injection, the ADA polymer solution turned turbid (Figure 3c), indicating a reaction triggered by CO_2_. Further microscopic characterization of the solution before and after CO_2_ treatment is shown in Figure 3d,e. Prior to CO_2_ injection, the ADA polymer formed a loose, entangled network in solution (Figure 3d). Following CO_2_ introduction, the electrostatic attraction provided by the quaternary ammonium and sulfonate groups strengthened both intramolecular and intermolecular physical crosslinking, resulting in a much denser network (Figure 3e). This electrostatically stabilized network can effectively trap water molecules, thereby increasing the solution viscosity and endowing the polymer with its distinctive CO_2_-triggered thickening behavior.

### 2.3. Rheological Properties of the ADA Polymer

**Figure 4:**
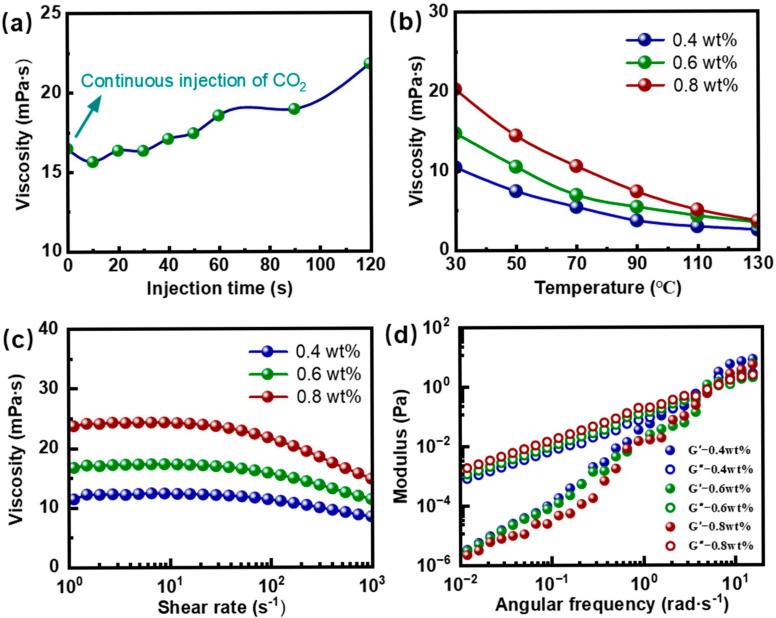
(**a**) The effect of CO_2_ injection time on the viscosity of ADA polymers. (**b**) The viscosity of the ADA polymer with different concentrations varies with temperature. (**c**) The viscosity of the ADA polymer with different concentrations varies with shear rate. (**d**) The viscoelastic modulus of ADA polymer with different concentrations varies with angular frequency.

The most prominent characteristic of the ADA polymer is its CO_2_-responsive viscosity enhancement. As shown in Figure 4a, the viscosity of 0.6 wt% ADA polymer solution increased from 16.47 mPa·s to 21.79 mPa·s upon 120 s of CO_2_ injection. This result confirmed the excellent CO_2_-triggered thickening ability of the polymer, indicating that gels constructed from ADA polymer possess potential CO_2_ resistance. Furthermore, the temperature resistance, shear stability, and viscoelasticity of ADA polymers at different concentrations were investigated. Figure 4b shows that with increasing polymer concentration, the number of polymer molecules in solution rises, enhancing the probability of intermolecular entanglement. This leads to a more stable network formed by chain entanglements, thereby improving the temperature resistance of the polymer solution.

During the injection of the conformance control agent into the formation, pore throats exert shear forces on the fluid. Therefore, the viscosity variations of ADA polymers at different concentrations (0.4 wt%, 0.6 wt%, and 0.8 wt%) under increasing shear rates were studied. As presented in Figure 4c, the viscosity decrease of the polymers with increasing shear rate was relatively small. When the shear rate increased from 1 s^−1^ to 100 s^−1^, the viscosity remained almost unchanged. Upon further increasing the shear rate to 1000 s^−1^, the viscosity of the 0.4 wt% ADA polymer solution decreased from 11.37 mPa·s to 8.34 mPa·s, the 0.6 wt% solution from 16.67 mPa·s to 11.27 mPa·s, and the 0.8 wt% solution from 23.66 mPa·s to 14.70 mPa·s. These results demonstrated the excellent shear resistance and low shear-thinning behavior of the ADA polymer. This performance is attributed to the rigid side chains provided by AMPS in the ADA polymer structure, which increase the steric hindrance within and between polymer chains. Under shear, this structure makes the polymer chains less prone to deformation and rearrangement, thereby reducing the impact of shear forces on viscosity [40]. The variations in the elastic modulus (G’) and viscous modulus (G″) with oscillatory frequency for ADA polymers at concentrations of 0.4 wt%, 0.6 wt%, and 0.8 wt% are shown in Figure 4d. For all concentrations, both G′ and G″ decreased with decreasing frequency. At lower frequencies, the viscous modulus exceeded the elastic modulus (G″ > G′). As the frequency increased, both G′ and G″ rose significantly, with the elastic modulus showing a more pronounced increase. Consequently, the gap between the two moduli narrowed, eventually leading to the elastic modulus surpassing the viscous modulus (G′ > G″). This behavior occurs because, under high-frequency oscillations, polymer chains cannot fully adjust their conformations for viscous flow. Instead, their inherent elasticity allows them to respond rapidly to external forces and store energy. Therefore, during the low-frequency shear process of polymer injection into the formation, the ADA polymer exhibits excellent viscous characteristics, enhancing injectability. Conversely, during the high-frequency shear encountered in plugging CO_2_ channels, the polymer demonstrates superior elastic properties, providing strong structural support.

### 2.4. CO_2_ Resistance and Rheological Properties of the ADA Gel

**Figure 5:**
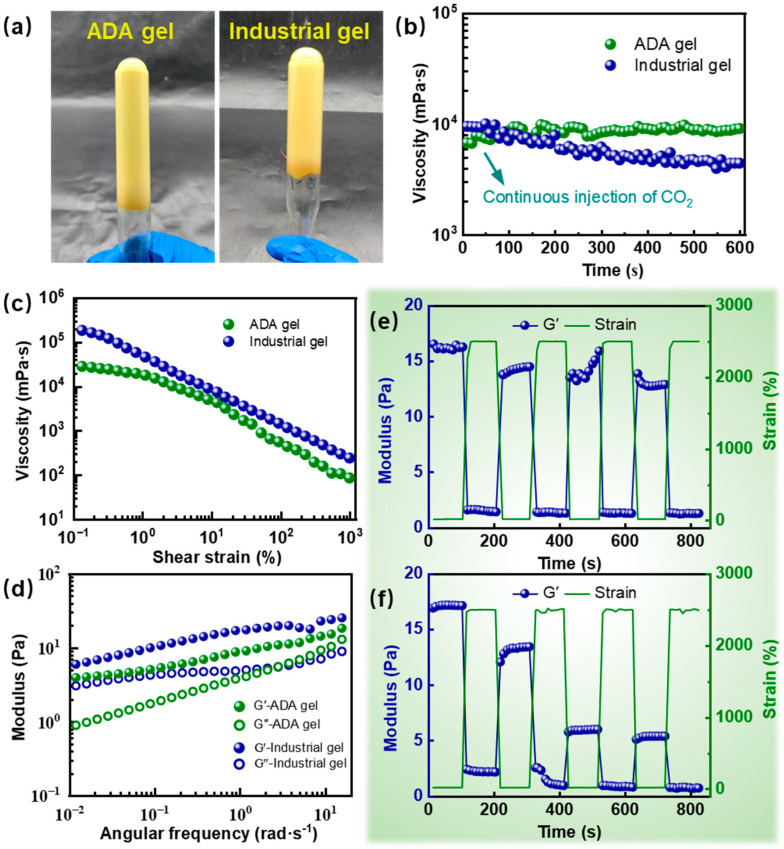
(**a**) Physical photos of ADA gel and industrial gel after gelation. (**b**) The effect of CO_2_ injection time on the viscosities of ADA gel and industrial gel. (**c**) The viscosities of ADA gel and industrial gel varies with shear strain. (**d**) The viscoelastic modulus of ADA gel and industrial gel varies with angular frequency, multi-cycle strain oscillation scanning curves of ADA gel (**e**) and industrial gel (**f**) after CO_2_ injection.

In this study, two types of gels were prepared: the ADA gel using 0.6 wt% ADA polymer and the industrial gel (control) using 0.6 wt% commercial FL polymer (mainly composed of HPAM), both crosslinked with 1.2 wt% highly reactive phenolic resin. As shown in Figure 5a, both polymers formed strong gels upon crosslinking, and their gel strengths visually evaluated by the GSC code method reached grade *H*, satisfying the strength requirement for gas channeling control.

As presented in Figure 5b, the initial viscosity of the ADA gel (7214 mPa·s) was lower than that of the industrial (9370 mPa·s). Under CO_2_ exposure, the viscosity of the conventional industrial gel continuously decreased with prolonged CO_2_ injection time. After 600 s of CO_2_ injection, its viscosity dropped to 4419 mPa·s, only 61% of the initial value. In contrast, the viscosity of the ADA gel increased to 9096 mPa·s after the same duration. These results demonstrate the excellent CO_2_ resistance of the ADA gel, indicating that CO_2_ does not degrade its gel strength. Furthermore, as shown in Figure 5c,d, in the absence of CO_2_, the industrial gel exhibited higher viscosity than the ADA gel over a shear rate range of 0.1 s^−1^ to 1000 s^−1^, and its moduli were generally superior to those of the ADA gel with increasing angular frequency. However, after CO_2_ treatment, multi-cycle shear recovery tests revealed that the storage modulus recovery rate of the ADA gel reached 78.61% (Figure 5e), whereas that of the industrial gel was only 31.24% (Figure 5f). This further confirms that the CO_2_ responsiveness of the ADA polymer imparts a more stable network structure and superior CO_2_ resistance to the gel. In contrast, the network of the industrial gel was significantly disrupted under combined CO_2_ and shear, leading to loss of stability.

**Figure 6:**
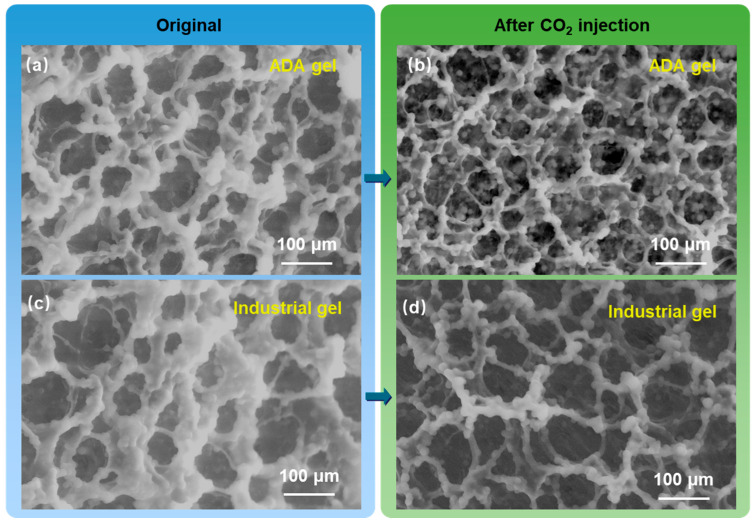
The ESEM images of the ADA gel and industrial gel before and after response to supercritical CO_2_, before CO_2_ injection (**a**,**c**), after CO_2_ injection (**b**,**d**).

The microstructures of both ADA gel and industrial gel before and after CO_2_ aging were further examined by ESEM to analyze the structural stability of the gel networks under a CO_2_ environment. As shown in Figure 6a,b, the presence of CO_2_ did not noticeably alter the network structure of the ADA gel, further confirming its excellent CO_2_ resistance and suitability for CO_2_ channeling control. In contrast, Figure 6c,d revealed that CO_2_ exposure caused the skeleton of the industrial gel to become thinner and introduced numerous voids, indicating severe network degradation. This deterioration occurs because CO_2_ dissolved in water creates a strongly acidic environment, which accelerates the hydrolysis of amide cross-linking sites and weakens the gel network. In the ADA polymer, CO_2_ protonates the tertiary amine groups to form positively charged quaternary ammonium salts. As illustrated in Figure 1b, these cationic side chains can shield the cross-linking sites from attack by H^+^ ions, thereby preserving the network. Moreover, the electrostatic supramolecular interactions between the positively charged quaternary ammonium groups and the negatively charged sulfonate groups in the CO_2_-responsive ADA polymer further enhance the cross-linked network strength. For these reasons, the constructed ADA polymer gel achieves CO_2_ tolerance and stability through its CO_2_-responsive architecture and a dense dual-crosslinked network.

### 2.5. Gas Channeling Control Capacity of the ADA Gel

**Figure 7:**
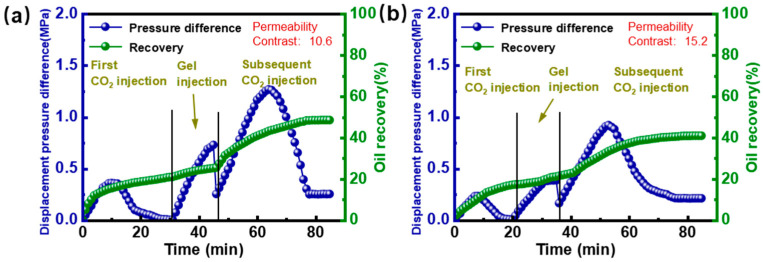
Changes in pressure difference and oil recovery during the whole CO_2_ injection process: (**a**) the core permeability contrast is 10.6, (**b**) the core permeability contrast is 15.2.

The Gas channeling control capacity and oil recovery of the ADA gel in heterogeneous cores were evaluated through core flooding experiments. The experiment involved injecting 1.0 pore volume (PV) of gel solution composed of 0.6 wt% ADA polymer and 1.2 wt% highly reactive phenolic resin crosslinker. As shown in Figure 7a, the permeability contrast of the core was 10.6, during the first CO_2_ flooding stage, as crude oil was produced, flow resistance decreased. After gas breakthrough occurred, the differential pressure dropped. The oil recovery at this stage was 20.8%, indicating that a significant amount of residual oil remained in the core. Subsequently, the gel solution was injected. Due to its higher viscosity, it improved the mobility ratio between the displacing CO_2_ and the crude oil, expanding the sweep volume. This stage increased oil recovery by 4.6%. After gelation, the second CO_2_ flooding was conducted. The gel formed a plugging barrier in the high-permeability layer, diverting CO_2_ to the low-permeability layer and mobilizing the oil there. This resulted in a significant oil recovery increase of 23.1%, demonstrating that the gel effectively enhances CO_2_ sweep efficiency and oil displacement. The study also found that increasing the permeability contrast (by raising the permeability of the high-permeability layer while keeping the low-permeability layer unchanged) reduces the overall oil recovery. As shown in Figure 7b, under higher permeability contrast conditions (15.2), the first CO_2_ flooding stage saw a 3.5% decrease in recovery due to earlier gas breakthrough. Compared with the core with a permeability contrast of 10.6, the ultimate recovery of the core with a permeability ratio of 15.2 was reduced from 48.5% to 40.9%. In summary, the CO_2_-resistant polymer gel can effectively plug high-permeability channels, promote the diversion of CO_2_ into low-permeability zones, and significantly improve oil recovery in heterogeneous cores.

## 3. Conclusions

A novel CO_2_-responsive polymer (ADA) was successfully synthesized and characterized. The incorporation of protonatable tertiary-amine groups and sulfonate moieties into the polymer backbone endowed the material with distinct CO_2_-responsive thickening, excellent shear resistance, and thermal stability suitable for reservoir conditions, laying a foundation for constructing CO_2_-resistant gels. The gel formulated from the ADA polymer exhibited superior CO_2_ resistance under simulated high-temperature and high-pressure reservoir environments. It maintained over 99% of its initial viscosity and a high storage-modulus recovery rate (78.61%) after prolonged CO_2_ exposure, significantly outperforming a conventional HPAM-based industrial gel (61% viscosity retention and 31.24% recovery rate). The enhanced CO_2_ tolerance of ADA gel was attributed to an electrostatically reinforced dual-crosslinked network. Protonation of tertiary amines generated cationic side chains that not only shielded the amide cross-linking sites from H^+^ attack but also engaged in electrostatic interactions with sulfonate groups, synergistically strengthening the gel network against both acidic and shear degradation. This gel system offers an effective technical solution for in-depth conformance control and storage-security enhancement in CO_2_ flooding of low-permeability reservoirs.

## 4. Materials and Methods

### 4.1. Materials

Acrylamide (AM, 99.9 wt%), dimethylaminopropyl methacrylamide (DMAPMA, 99 wt%), 2-acrylamide-2-methyl-1-propanesulfonic acid (AMPS, 98 wt%) and azodiisopropylimidazoline hydrochloride (AIBI, 96 wt%) were purchased from Shanghai McLean Biochemical Co., Ltd., Shanghai, China. High-activity phenolic resin crosslinking agent was purchased from Shandong Lifeng Chemical Co., Ltd., Jinan, China. All reagents were used as received without further purification. The industrial polymer used as the control group was FL polymer, which was purchased from Hebei Langfang Xinxing Chemical Co., Ltd., Langfang, China. Deionized water was made in the laboratory (18.25 MΩ·cm). The crude oil was collected from the Tarim Oilfield with a density of 0.834 g·cm^−1^ and a viscosity of 35.61 mPa·s (110 °C). The artificial sandstone core was provided by Haian Petroleum Research Instrument Co., Ltd., Haian, China. The core parameters are shown in Table 1.

### 4.2. Preparation of CO_2_-Responsive Polymer

The CO_2_-responsive polymer was synthesized via solution polymerization following the scheme illustrated in Figure 1a. The detailed synthesis procedure is described as follows. First, 24.75 g of AM, 1.5 g of DMAPMA, and 3.75 g of AMPS were dissolved in 69 g of deionized water under stirring. The pH of the solution was then adjusted to 7.0. The reaction mixture was transferred into a three-necked flask and heated to 60 °C. Dissolved oxygen was removed by purging the system with nitrogen, after which 1.0 g of AIBI was added as the initiator to start the polymerization. Upon completion of the reaction, the polymer product was granulated and vacuum-dried in an oven, finally obtaining the CO_2_-responsive polymer designated as ADA.

### 4.3. Preparation of CO_2_-Resistant Gel

The CO_2_-resistant gel was formed by crosslinking the CO_2_-responsive polymer (ADA) with a high-activity phenolic resin crosslinking agent, with the resulting crosslinked structure illustrated in Figure 1b. The specific preparation procedure is as follows. First, 0.6 wt% of the CO_2_-responsive polymer (ADA) was dissolved in water under continuous stirring. Then, 1.2 wt% of a highly reactive phenolic resin crosslinker was added and stirred until completely dissolved. The resulting homogeneous solution was transferred into an ampoule, sealed, and placed in an oven at 110 °C for 2 days to form the final CO_2_-resistant gel.

### 4.4. Chemical Structure Characterization

Fourier-transform infrared (FT-IR) spectroscopy was performed using a Nicolet IS50 spectrometer (Thermo Fisher Scientific, Waltham, MA, USA) to analyze the functional groups of the synthesized polymer. X-ray diffraction (XRD) analysis was conducted on a D8 Advance X diffractometer (Bruker, Karlsruhe, Germany) to examine the crystalline structure. Measurements were operated at 40 kV and 30 mA. The scanning range was set from 5° to 90° (2θ) with a scan speed of 5°·min^−1^. Thermal stability of the polymer was evaluated by thermogravimetric analysis (TGA) on a TG 209 F3 instrument (Netzsch, Schlierbach, Germany). Approximately 15 mg of the polymer sample was placed in an alumina crucible and heated from 35 °C to 800 °C under a nitrogen atmosphere at a constant heating rate of 10 °C·min^−1^.

### 4.5. Microstructure Characterization of the Polymers and Gels

The microscopic morphology and network structure of the polymers and gels were analyzed using a Quanta 200 environmental scanning electron microscope (ESEM, FEI Company, Portland, OR, USA) equipped with a Peltier cooling stage. The ESEM enables direct observation of samples in their natural state without the need for dehydration or gold coating, making it particularly suitable for polymer gel specimens. The experimental conditions were set as follows: temperature of 2 °C, chamber pressure of 400 Pa, and an accelerating voltage of 10 kV during imaging.

### 4.6. Rheological Testing of the Polymers and Gels

The rheological properties of the polymer and gel were measured using a Haake Mars 60 high-temperature/high-pressure rheometer (Thermo Fisher Scientific, Waltham, MA, USA). A Hastelloy high-pressure cylindrical measurement cell, resistant to high temperature, high pressure, and acidic corrosion, was employed for testing.

① Rheological testing of the polymers

A constant shear rate of 7.34 s^−1^ was set to determine the viscosity of the polymer under reservoir conditions. Shear stability was evaluated by varying the shear rate from 0.01 s^−1^ to 1000 s^−1^ under simulated reservoir conditions. Stress sweep tests were first performed at a fixed angular frequency of 6.28 rad·s^−1^ (1.0 Hz) to identify the linear viscoelastic region. A stable shear stress within this region was then selected for frequency sweep tests, which were conducted over an angular frequency range of 0.01–100 rad·s^−1^ at 25 °C. Furthermore, the viscoelastic behavior of the polymer under varying shear rates was investigated by scanning from 0.01 s^−1^ to 1000 s^−1^.

② Rheological testing of the gels

The testing conditions and methods for evaluating the viscoelastic properties and shear resistance of the gel were similar to those used for the polymer. In addition, multi-cycle variable-strain oscillatory shear tests on the gel were conducted using a parallel-plate system. A high shear strain (2500%) was applied for 100 s, and the stabilized storage modulus was recorded. This was followed by a low shear strain (10%) applied for 100 s, with the corresponding stabilized storage modulus recorded. The storage modulus recovery rate (*R*) of the gel was calculated using Equation (1) [41], as follows:

(1) $$R=\frac{{G_{1}}^{\prime }}{{G_{2}}^{\prime }}\times 100\%$$

where *R* is the storage modulus recovery rate (%), *G*_1_*’* is the stabilized storage modulus under low strain in the last cycle (Pa), and *G*_2_*’* is the initial stabilized storage modulus under low strain (Pa).

### 4.7. Gas Channeling Control Experiment

First, the dehydrated and degassed crude oil and the fractured core were vacuumed and then pressurized to saturate with crude oil. The core was then aged in an oven at 110 °C for 2 days, and the core parameters are shown in Table 1. After aging, the core was loaded into a core holder, and the confining pressure was maintained 3.0 MPa above the injection pressure throughout the experiment. CO_2_ was injected into the core at a constant flow rate of 0.20 mL/min for the first CO_2_ flooding. Subsequently, a specific volume of polymer gel solution was injected into the core at the same flow rate of 0.20 mL/min. The core holder was then sealed and placed in a constant-temperature oven at 110 °C until complete gelation was achieved. Following gelation, CO_2_ was injected again into the core at 0.20 mL/min for the second CO_2_ flooding. During the experiment, the differential pressure and production at the outlet were continuously recorded.

## Figures and Tables

**Table 1:** Core parameters.

Number	Length/mm	Diameter/mm	Permeability/10^−3^ μm^2^		Porosity/%		Permeability Contrast
			High	Low	High	Low	
1	100.42	24.59	107.71	10.12	17.12	12.73	10.6
2	100.28	24.75	140.90	9.27	13.31	10.46	15.2

## Data Availability

The original contributions presented in the study are included in the article, further inquiries can be directed to the corresponding authors.
